# Macrophage Activation in Pediatric Nonalcoholic Fatty Liver Disease (NAFLD) Correlates with Hepatic Progenitor Cell Response via Wnt3a Pathway

**DOI:** 10.1371/journal.pone.0157246

**Published:** 2016-06-16

**Authors:** Guido Carpino, Valerio Nobili, Anastasia Renzi, Cristiano De Stefanis, Laura Stronati, Antonio Franchitto, Anna Alisi, Paolo Onori, Rita De Vito, Gianfranco Alpini, Eugenio Gaudio

**Affiliations:** 1 Department of Movement, Human and Health Sciences, University of Rome "Foro Italico", Rome, Italy; 2 Unit of Liver Research, Bambino Gesù Children's Hospital, Rome, Italy; 3 Department of Anatomical, Histological, Forensic Medicine and Orthopedics Sciences, Sapienza University of Rome, Rome, Italy; 4 Department of Radiobiology and Human Health, ENEA, Rome, Italy; 5 Unit of Pathology, Bambino Gesù Children's Hospital, Rome, Italy; 6 Research, Central Texas Veterans Health Care System, Department of Medicine, Scott & White Digestive Disease Research Center, Scott & White and Texas A&M Health Science Center College of Medicine, Temple, Texas, United States of America; Institute of Hepatology, Foundation for Liver Research, UNITED KINGDOM

## Abstract

Non-alcoholic fatty liver disease is one of the most important causes of liver-related morbidity in children. In non-alcoholic fatty liver disease, the activation of liver resident macrophage pool is a central event in the progression of liver injury. The aims of the present study were to evaluate the polarization of liver macrophages and the possible role of Wnt3a production by macrophages in hepatic progenitor cell response in the progression of pediatric non-alcoholic fatty liver disease. 32 children with biopsy-proven non-alcoholic fatty liver disease were included. 20 out of 32 patients were treated with docosahexaenoic acid for 18 months and biopsies at the baseline and after 18 months were included. Hepatic progenitor cell activation, macrophage subsets and Wnt/β-catenin pathway were evaluated by immunohistochemistry and immunofluorescence. Our results indicated that in pediatric non-alcoholic fatty liver disease, pro-inflammatory macrophages were the predominant subset. Macrophage polarization was correlated with Non-alcoholic fatty liver disease Activity Score, ductular reaction, and portal fibrosis; docosahexaenoic acid treatment determined a macrophage polarization towards an anti-inflammatory phenotype in correlation with the reduction of serum inflammatory cytokines, with increased macrophage apoptosis, and with the up-regulation of macrophage Wnt3a expression; macrophage Wnt3a expression was correlated with β-catenin phosphorylation in hepatic progenitor cells and signs of commitment towards hepatocyte fate. In conclusion, macrophage polarization seems to have a key role in the progression of pediatric non-alcoholic fatty liver disease; the modulation of macrophage polarization could drive hepatic progenitor cell response by Wnt3a production.

## Introduction

Non-alcoholic fatty liver disease (NAFLD) is one of the most important causes of liver-related morbidity in children [1]. The development of definite steatohepatitis (NASH) is determined by intricate interactions between resident and recruited cells [2]. In adult NAFLD, the activation of liver resident macrophage pool is a central event in the initiation and progression of liver injury [3, 4]. Macrophages have the ability to change their activation states in response to growth factor and external stimuli [5]. Functional subdivisions have been proposed in accordance with a spectrum of activation states [6, 7]. Activated macrophages can produce pro-inflammatory cytokines and have pivotal role in inflammatory response; besides, macrophages with an anti-inflammatory phenotype are involved in tissue repair and efficient phagocytosis of cellular debris [6].

Liver macrophages are a key component of hepatic progenitor cell (HPC) niche, regulating their activation and fate choice [8]. The HPC activation takes part in regeneration after liver injury [9, 10] and is involved in the progression of pediatric NAFLD (pNAFLD) [11].

Recently, N-3 long-chain polyunsaturated fatty acids (LC-PUFA) supplementation has been suggested as a potential treatment for liver steatosis [12, 13]. The effects of docosahexaenoic acid (DHA), the major dietary LC-PUFA, have been reported in pNAFLD [14, 15]. Interestingly, DHA exerts a potent anti-inflammatory activity on macrophages [16].

The aims of the present study are to evaluate i) the activation states of liver macrophages in pNAFLD and the correlation with the progression towards NASH and with HPC response; ii) if the DHA administration in pediatric patients induces modifications on macrophage activation; and iii) the role of Wnt3a macrophage production on HPC response in pNAFLD.

## Patients and Methods

### Patients

This study included 32 children and adolescents (boys, 22; girls, 10; median age, 10.8 years; range 7–16 years) with biopsy-proven NAFLD, who were referred to Bambino Gesù Children’s Hospital during January 2010-January 2013.

20 out of 32 patients have been enrolled from the randomized controlled clinical trial registered on http://www.clinicaltrials.gov (**Trial identifier:** NCT00885313) conducted at the Liver Unit of the Bambino Gesù Pediatric Hospital (Rome, Italy) and received algae DHA (250 mg/day) for 18 months [14, 15]. Twelve patients were not included in the clinical trial, did not receive DHA supplementation and were treated by standard lifestyle intervention program.

All patients included in the present study met the following criteria: persistently elevated serum alanine transaminase (ALT ≥40 U/l), diffusely hyperechogenic liver at ultrasonography and liver biopsy consistent with NAFLD, as previously reported [17]. Patients with secondary causes of steatosis were excluded from the trial and this entire study. The children included in our analyses showed clinical and pathological features resembling those seen in our general pediatric population with NAFLD [18]. Patients were diagnosed with NAFLD through liver biopsy recommended because of over 6 months elevation of ALT levels and the presence of an echogenic texture of the liver on ultrasonography. Patients received no dietary or other therapeutic treatment regimens before diagnosis. Clinical data were acquired at diagnosis and after 18 months of treatment. Liver biopsy was taken at the diagnosis and after 18 months in patients treated with DHA. For ethical reasons, liver biopsy was taken at the diagnosis but not after 18 months in control patients who underwent lifestyle intervention program [19].

Liver specimens from 6 lean, non-diabetic children (boys, 4; girls, 2; median age: 13 years, range, 12–16 years) without liver disease were used as controls, as previously [11]. These fragments were obtained from patients who underwent laparotomy or laparoscopic procedures (for cholecystectomy), from liver donors (orthotopic liver transplantation) or incidental “normal” liver biopsies (children exhibiting persistent/intermittent elevations of liver enzymes for >6 months). Informed consent in writing was obtained from next of kin, caretakers, or guardians on behalf of the children enrolled in this study. The study protocol conformed to the ethical guidelines of the 1975 Declaration of Helsinki as reflected in a priori approval by the Bambino Gesù Pediatric Hospital ethics committee. No donor organs were obtained from any vulnerable populations, aside from being children. Anthropometric (Weight, height, and body mass index) and laboratory data were measured as previously reported [18].

### Anthropometrics and laboratory data

Weight, height, and body mass index (BMI) were measured. Alanine and aspartate aminotransferase, gamma-glutamyl-transpeptidase, total triglycerides and total cholesterol were evaluated using standard laboratory methods. Insulin was measured using a radio-immunoassay (Myria Technogenetics, Milan, Italy). Glucose and insulin were measured at 0, 30, 60, 90 and 120 minutes of an oral glucose tolerance test performed with 1.75 grams of glucose per kilogram of body weight (up to 75g). The degree of insulin resistance and sensitivity were determined by the homeostatic model assessment insulin resistance (HOMA-IR) and the insulin sensitivity index (ISI) derived from an oral glucose tolerance test (OGGT), respectively [20, 21].

### Liver Biopsy and Histo-pathological Analysis

Liver biopsy was performed after an overnight fast by using an automatic core biopsy 18-gauge needle under general anesthesia and ultrasound guidance. Specimens were fixed in formalin and embedded in paraffin, as previously reported [17]. Standard histological stains were performed. Histopahtological evaluation has been performed on the basis of the NAFLD Clinical Research Network (CRN) criteria [22]. The NAFLD activity score (NAS) has been calculated combining features of steatosis, lobular inflammation, and hepatocyte ballooning. As recommended [23], a microscopic diagnosis based on overall injury pattern as well as the presence of additional lesions have been assigned to each case [24]. Biopsies were classified into not steatohepatitis (NAFL), definite steatohepatitis (NASH), borderline zone 1 pattern or borderline zone 3 pattern subcategories [23].

Histological analysis was performed by a single pathologist blinded to clinical and laboratory data.

### Immunohistochemistry and Immunofluorescence

For immunohistochemistry and immunofluorescence, sections were incubated overnight at 4°C with primary antibodies against cytokeratin (CK)7 (mouse monoclonal, code: M7018, dilution: 1:100, Dako, Glostrup, Denmark), EpCAM (Dako, mouse monoclonal, code: M3525, dilution: 1:100), CD68 (Dako, mouse monoclonal, code: M0876, dilution: 1:100), CD206 (monoclonal mouse, code: MAB25341; dilution: 1:25, R&D Systems, Minneapolis, USA), Arginase-1 (polyclonal mouse, code: ab118884; dilution: 1:150, Abcam, Cambridge, United Kingdom), Caspase-3 (polyclonal rabbit, code: #9662; dilution: 1:200, Cell Signaling Technology, Danvers, USA), CD163 (monoclonal mouse, code: ncl-cd163; dilution: 1:200, Novocastra, Milan, Italy), S100A9 (polyclonal rabbit, code: ab92507; dilution: 1:200, Abcam, Cambridge, United Kingdom), Wnt3a (polyclonal rabbit, code: #09–162; dilution: 1:200, Merck Millipore, Darmstadt, Germany), SOX9 (polyclonal rabbit, code: AB5809; dilution: 1:200, Millipore, Darmstadt, Germany), and phosphorylated (p) β-catenin (Cell Signaling Technology, rabbit polyclonal, code: #4176, dilution 1:100). For immunohistochemistry, samples were than incubated for 20 minutes at room temperature with secondary biotinylated antibody and, successively, with streptavidin-Horse radish peroxidase (LSAB+, Dako, code K0690). Diaminobenzidine (Dako, code K3468) was used as the substrate and the sections were counterstained with hematoxylin or Sirius Red [25].

For immunofluorescence, non-specific protein binding was blocked with 5% normal goat serum. Sections were incubated with primary antibodies, and subsequently incubated with labeled isotype-specific secondary antibodies (anti-mouse AlexaFluor-488 and anti-rabbit Alexafluor-594, Invitrogen Ltd, Paisley, UK) for 1 hour; nuclei were visualized with 4,6-diamidino-2-phenylindole (DAPI) [17].

To perform double immunostaining with two mouse or rabbit primary antibodies (CD68/CD206), we followed a 3-step protocol [11, 26]: sections were incubated with anti-CD68 (or anti-SOX9), an anti-mouse (or anti-rabbit) secondary fluorescent antibody (alexafluor-488) was applied, and the second primary antibody was pre-labeled with a fluorophore using APEX-594 labeling Kit (Invitrogen) and applied to the section. All antibodies were diluted (1:50) and incubated for 1 hour. Slides were counterstained with 4’,6-diamidino-2-phenylindole (DAPI). For all immunoreactions, adequate negative controls were also preformed.

Sections were examined with a Leica Microsystems DM 4500 B Microscopy (Weltzlar, Germany) equipped with a Jenoptik Prog Res C10 Plus Videocam (Jena, Germany) and with an Olympus Fluoview FV1000 confocal microscope equipped with FV10-ASW version 4.1 software. Only biopsies containing at least 5 portal spaces were considered [27].

The extension of ductular reaction (DR) was evaluated by CK7 immunoreactivity. CK7 stained slides were scanned by a digital scanner (Aperio Scanscope CS System, Aperio Digital Patology, Leika Biosystems, Milan, Italy) and processed by ImageScope [11, 17]. The area occupied by CK7+ cells was quantified by an image analysis algorithm. The extension of DR was expressed as the percentage of the parenchymal area occupied by reactive ductules [28]. Cholangiocytes lining the interlobular bile ducts were excluded from the counts.

To assess the commitment of progenitor cells toward a hepatocyte fate, the presence of EpCAM+ hepatocytes has been investigated by immunohistochemistry. EpCAM+ hepatocytes have been shown to represent the progeny of stem/progenitor cells within bile ductules [29, 30]. The presence of EpCAM+ hepatocytes was scored as: 0 = no positive cells, 1 (level 1) = single occasional, and 2 (level 2) = clusters of EpCAM+ hepatocyte [29, 31].

The extension of portal fibrosis was evaluated on Sirius Red stains and the area occupied by Sirius Red-positive fibers in the entire section was quantified as previously [32].

The number of macrophages with an anti-inflammatory phenotype was calculated as the number of CD206+ cells per High Powered Field (HPF) [4, 33]. In DHA treated patients, Arginase1 and CD163 have been further used as markers of anti-inflammatory macrophages. The presence of macrophages with an inflammatory phenotype was calculated as the number of S100A9+ cells per High Powered Field (HPF) [4, 33, 34].

Wnt3a expression by CD68+ macrophages was evaluated in serial sections and by double immunofluorescence as the number of positive macrophages per HPF. pβ-catenin expression by CK7+ HPCs was evaluated in serial sections and by double immunofluorescence; the average number of positive cells was divided by the average number of HPCs and data were expressed as a percentage of positive cells [11]. Given the differences in term of DR extension among examined biopsies, pβ-Catenin-positivity was further calculated as the ratio between the extension of positive DR (by ImageScope, Aperio) and the total DR extension.

For confocal microscopy imaging, fluorochrome unmixing was performed by acquisition of automated-sequential collection of multi-channel images, in order to reduce spectral crosstalk between channels. The average number of cells that displayed a colocalization of Caspase-3/CD68 or Arginase1/CD163 was assessed by counting 5 fields acquired using 20x.

### Cytokine Assay

Cytokine-specific ELISA assays were used to determine serum levels of pro-inflammatory cytokines, as previoulsy reported [17]. Concentrations were evaluated by manufacturing protocols from RayBiotech Inc (Norcross GA, USA) for Interleukin (IL)-1β and IL-6 and from Immundiagnostik (AG, Bensheim, Germany) for tumour necrosis factor (TNF)-α.

### Statistical methods

Data are indicated as median [25^th^ percentile, 75^th^ percentile]. The nonparametric Mann—Whitney U test was used to compare two groups. To evaluate the modification of variables after DHA treatment, the Wilcoxon matched-pairs signed rank test was applied. The Spearman nonparametric correlation was used. A *p*-value of <0.05 was considered statistically significant. Statistical analyses were performed using SPSS statistical software (SPSS Inc. Chicago IL, USA).

## Results

### Anthropometrics laboratory data and Histo-pathology evaluation

We included 32 patients in this study. Twelve patients underwent lifestyle intervention program (Table 1).

**Table 1:** Anthropometrics and laboratory data of twelve patients affected by NAFLD and who underwent lifestyle intervention program and did not receive DHA administration.

	*T0 (N = 12)*	*T1 (N = 12)*	*p*
Age (years)	12 [9.95, 13.9]	12.7 [10.44, 14]	0.90
BMI (kg/mq)	26.07 [24.36, 28.99]	25.04 [23.3, 28]	0.38
WC (cm)	62 [58.15, 76.2]	58.2 [52.35, 71.3]	0.39
z-BMI	1.99 [1.59, 2.78]	1.82 [1.36, 2.21]	0.29
AST (UI/L)	54 [41.25, 64.75]	43 [37.7, 46.25]	**0.001**
ALT (UI/L)	79 [67, 90]	50 [42.5, 56.75]	**0.001**
GGT (UI/L)	23.9 [20.75, 27.25]	25.4 [21.5, 27]	0.74
Total Cholesterol (mg/dl)	160.5 [142.5, 171.5]	154 [135.5, 166.7]	0.86
LDL Cholesterol (mg/dl)	106 [89.35, 114.75]	93.6 [74.4, 101]	0.25
HDL Cholesretol (mg/dl)	36 [28.75, 44]	41 [32.5, 46]	0.49
Triglycerides (mg/dl)	94 [78.5, 112]	94 [86.75, 102]	0.91
Fasting plasma glucose (mg/dl)	87 [81.75, 90]	82.35 [4.21]	0.07
Fasting plasma gluc-120’	102 [96, 110]	95[82.7, 98]	0.08
Insulin (mU/L)	11.15 [9, 15]	9.8[7.52, 13.2]	0.10
Insulin -120’	98 [66.5, 121.4]	95 [88, 101]	0.10
HOMA-IR	2.40 [1.96, 3.35]	2.01 [1.58, 2.74]	0.10
ISI	3.65 [3.12, 5]	3.1 [2.57, 4.7]	0.05

Data are indicated as Median [25^th^ percentile, 75^th^ percentile]. *p values* < 0.05 are in **bold**. T0 = baseline; T1 = at the end of treatment.

Besides, 20 patients were enrolled in the aforementioned trial and treated with DHA for 18 months (Table 2).

**Table 2:** Anthropometrics, laboratory and histological data of NAFLD patients who received DHA supplementation for 18 months.

	T0 (N = 20)	T1 (N = 20)	p
Age (years)	10.9 [9.49, 12]	12 [10.5, 13.7]	0.10
BMI (kg/mq)	25.5 [22.9, 27.1]	25 [21.9, 26.2]	0.10
WC (cm)	65 [60, 68.7]	62.4 [58.3, 65.5]	0.11
z-BMI	2.23 [1.98, 2.79]	2.04 [1.94, 2.63]	0.42
AST (UI/L)	57 [35.7, 67]	34 [31, 40]	**<0.001**
ALT (UI/L)	65 [47.7, 75]	35 [31, 40]	**<0.001**
GGT (UI/L)	21 [14, 27.2]	21.5 [17.7, 24]	0.21
Total Cholesterol (mg/dl)	162 [134.7, 166]	160 [132, 165]	0.72
LDL Cholesterol (mg/dl)	77.5 [69, 101]	63 [49.7, 80.6]	**0.005**
HDL Cholesretol (mg/dl)	52.5 [40.5, 64.7]	41 [35.7, 51.2]	0.44
Triglycerides (mg/dl)	89.5 [77.7, 102]	78 [62.2, 90.2]	**0.04**
Fasting plasma glucose (mg/dl)	84.5 [78.7, 90]	79 [74.7, 81.5]	0.32
Fasting plasma gluc-120’	112.8 [100, 122.6]	101 [94.7, 113.2]	0.39
Insulin (mU/L)	14 [7.27, 19.4]	9.6 [5.7, 11.3]	**0.04**
Insulin -120’	121 [101, 134.5]	61.2 [46.7, 107.9]	**<0.001**
HOMA-IR	2.19 [1.56, 3.6]	1.61 [1, 1.96]	0.05
ISI	3.63 [3.1, 5.3]	5.32 [4.8, 6.8]	**0.04**
Steatosis	2 [1,2]	0[0,1]	**<0.001**
Ballooning	1 [1,1]	0 [0,1]	**0.02**
Portal Inflammation	2 [1,2]	1[0.75,1]	0.05
Lobular Inflammation	1[1,2]	1[0,1]	0.05
Fibrosis	2 [1,2]	2[1,2]	0.54
NAS	4[3,4]	2[1,2]	**0.01**

Data are indicated as Median [25^th^ percentile, 75^th^ percentile]. *p values* < 0.05 are in **bold**. T0 = baseline; T1 = at the end of treatment.

With regard to anthropometric and laboratory data (Table 3), ALT, AST, HDL cholesterol, Triglycerides, fasting plasma glucose– 120’, and ISI were significantly improved after DHA treatment in comparison with patients who did not receive DHA.

**Table 3:** Comparison of anthropometrics and laboratory data between NAFLD patients who did not receive DHA supplementation (T1—NAFLD) and NAFLD patients who received DHA supplementation (T1—DHA).

	T1 –NAFLD (N = 12)	T1 –DHA (N = 20)	*p*
Age (years)	12.7 [10.44, 14]	12 [10.5, 13.7]	0.54
BMI (kg/mq)	25.04 [23.3, 28]	25 [21.9, 26.2]	0.22
WC (cm)	58.2 [52.35, 71.3]	62.4 [58.3, 65.5]	0.68
z-BMI	1.82 [1.36, 2.21]	2.04 [1.94, 2.63]	0.11
AST (UI/L)	43 [37.7, 46.25]	34 [31, 40]	**0.02**
ALT (UI/L)	50 [42.5, 56.75]	35 [31, 40]	**0.001**
GGT (UI/L)	25.4 [21.5, 27]	21.5 [17.7, 24]	0.10
Total Cholesterol (mg/dl)	154 [135.5, 166.7]	160 [132, 165]	0.09
LDL Cholesterol (mg/dl)	93.6 [74.4, 101]	63 [49.7, 80.6]	**0.001**
HDL Cholesretol (mg/dl)	41 [32.5, 46]	41 [35.7, 51.2]	**0.03**
Triglycerides (mg/dl)	94 [86.75, 102]	78 [62.2, 90.2]	**0.002**
Fasting plasma glucose (mg/dl)	82.35 [4.21]	79 [74.7, 81.5]	0.92
Fasting plasma gluc-120’	95 [82.7, 98]	101 [94.7, 113.2]	**0.04**
Insulin (mU/L)	9.8 [7.52, 13.2]	9.6 [5.7, 11.3]	0.27
Insulin -120’	95 [88, 101]	61.2 [46.7, 107.9]	0.81
HOMA-IR	2.01 [1.58, 2.74]	1.61 [1, 1.96]	0.05
ISI	3.1 [2.57, 4.7]	5.32 [4.8, 6.8]	**0.01**

Data are indicated as Median [25^th^ percentile, 75^th^ percentile]. *p values* < 0.05 are in **bold**. T1 = at the end of treatment.

Liver biopsies were classified into: NAFL (simple steatosis; N = 8), NASH (definite steatohepatitis, N = 19), borderline zone1 pattern (N = 1), and borderline zone3 pattern (N = 4). The NAS scores ranged from 1 to 7. Fibrosis of some degree was seen in all biopsy samples: stage 1c in 13 samples, stage 2 in 18, and stage 3 in 1. Normal liver samples had normal histological features.

### HPCs in pNAFLD biopsies

**Fig 1:**
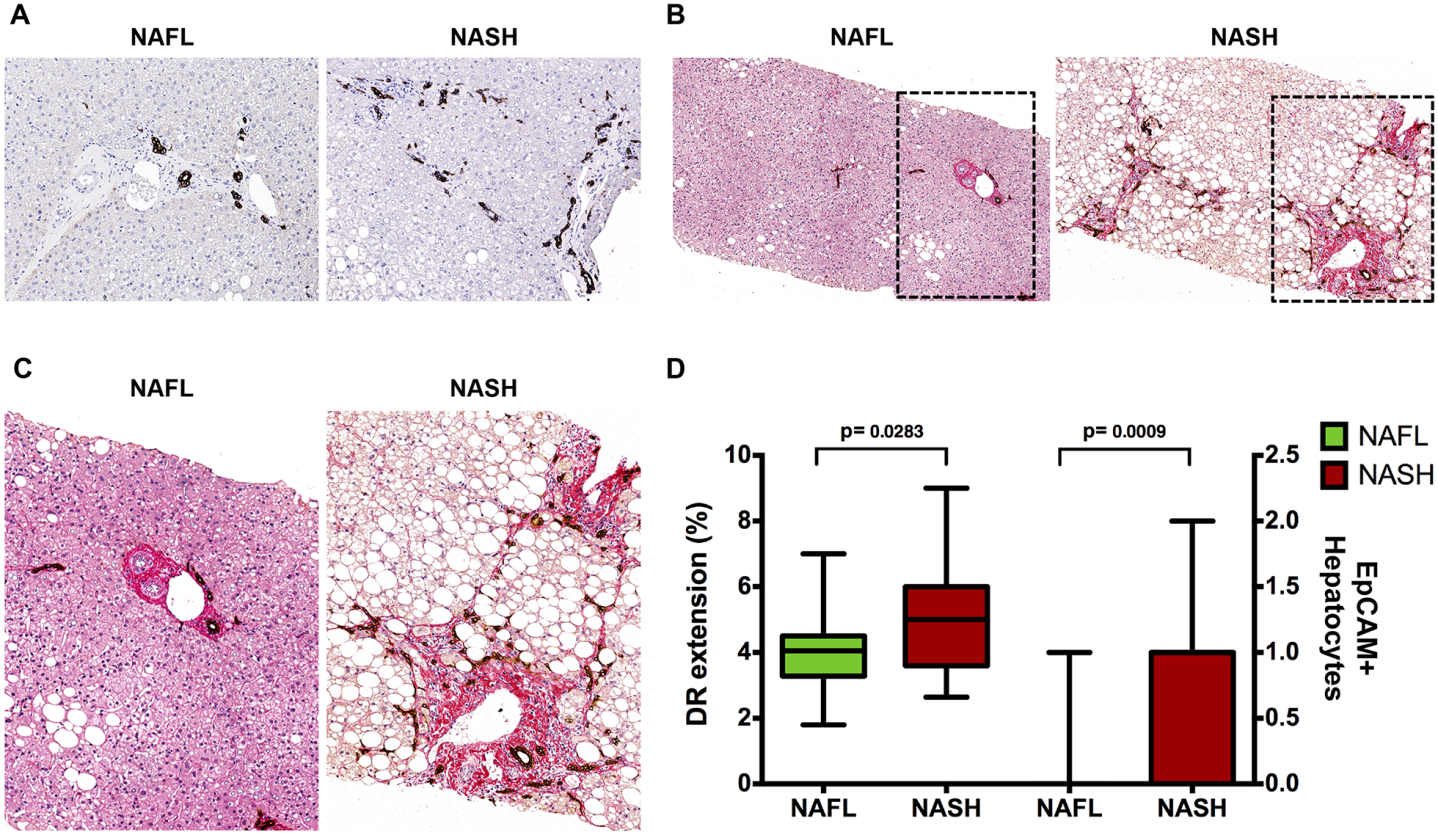
Ductular Reaction (DR) and portal fibrosis in pediatric NAFLD. A) Immunohistochemistry for cytokeratin (CK)7 in pediatric NAFLD biopsies. DR extension is increased in definite steatohepatitis (NASH) in comparison with not-SH (NAFL). Original Magnification (OM) = 10x. B-C) Immunohistochemistry for CK7 is counterstained with Sirius Red. DR extension is associated with portal fibrosis. OM = 10x (B) and 20x (C). (D) Box-and-Whisker Plots (median, quartile ranges, minimum—maximum) regarding DR and EpCAM+ hepatocytes.

The degree of HPC activation was evaluated by the extension of DR and the presence of IHs was considered as a sign of HPC commitment towards hepatocyte fate [11]. Overall, pNAFLD samples showed an evident DR and the area occupied by DR accounted for 4.20 [3.32, 5.35] percent of liver parenchyma; in normal livers, DR was not present and the area occupied by bile ductules was lower in comparison with DR extension in pNAFLD (1.90 [1.35, 2.05]; p<0.01). When histological classification was taken in consideration (Fig 1A), NASH biopsies presented a higher DR expansion (median = 5.00 [3.60, 6.00]) compared with NAFL samples (median = 4.05 [3.28, 4.50], p<0.05); values in the latter, however, were higher than those in normal livers (p< 0.05). EpCAM+ hepatocytes were not found in NAFL biopsies (0/8); contrarily, single occasional or clusters of EpCAM+ hepatocytes were present in 14/19 (73%) NASH biopsies. The presence of EpCAM+ hepatocytes was correlated with DR (r = 0.611, p<0.001). Both DR extension and the presence of EpCAM+ hepatocytes were significantly correlated with NAS score (r = 0.498 and r = 0.442, respectively; p<0.01). Finally, DR extension was strongly correlated with portal fibrosis extension (r = 0.719, p<0.01; Fig 1B and 1C).

### Macrophage activation in pNAFLD biopsies

Overall, in pNAFLD biopsies, the number of total (CD68+) macrophages was increased (median: 24.4 [17.35, 31.43]) in comparison with normals (median: 15.80 [13.70, 17.95], p<0.05, Table 4).

**Table 4:** Macrophage number and phenotype in liver biopsies obtained from normal subjects and in pediatric patients affected by NAFLD.

	Normal (N = 6)	NAFLD (N = 32)	NAFL (N = 8)	NASH (N = 19)
**CD68+ Mϕ**	15.8 [13.7, 17.95]	24.4 [17.35, 31.43]*	17.30 [14.25, 23.60]	28.80 [21.80, 36.50]#
**S100A9+ Mϕ**	5.00 [4.00, 6.00]	13.00 [7.25, 15.0]*	7.00 [7.00, 10.50]*	15.00 [13.00, 15.00] #
**CD206+ Mϕ**	15.10 [12.50, 16.70]	9.90 [7.67, 11.25]*	11.00 [9.91, 14.25]	9.15 [7.32, 10.13]#
***Lo* CD68+ Mϕ**	11.20 [8.85, 11.65]	11.15 [9.47, 15.95]	9.30 [9.00, 11.10]	14.00 [10.30, 19.50]#
***Lo* S100A9+ Mϕ**	4.00 [3.50, 5.00]	8.50 [6.00, 11.00]*	6.00 [5.00, 6.00]*	11.00 [9.00, 12.00]#
***Lo* CD206+ Mϕ**	10.60 [8.80, 13.85]	6.00 [3.92, 7.56]*	6.50 [4.50, 9.91]*	5.30 [3.52, 7.00]*
***Po* CD68+ Mϕ**	3.95 [3.12, 5.72]	11.00 [7.62, 14.75]*	8.0 [5.25, 10.00]*	14.00 [10.00, 15.70]*
***Po*S100A9+ Mϕ**	1.00 [0, 1.00]	5.50 [5.00, 9.00]*	5.00 [4.00, 5.50]*	6.00 [5.00, 10.00]
***Po* CD206+ Mϕ**	3.50 [2.60, 4.45]	4.00 [3.00, 5.00]	4.50 [4.00, 5.00]	4.00 [2.75, 4.00]

Data are indicated as Median [25^th^ percentile, 75^th^ percentile].

* = p<0.05 versus Normal;

^#^ = p<0.05 versus NAFL and Normal.

Mϕ = macrophages; lo = lobular; po = portal. NAFLD biopsies were further classified in accordance with overall diagnosis in Non-alcoholic Fatty Liver (NAFL or simple steatosis) and in Non-alcoholic Steato-hepatitis (NASH).

**Fig 2:**
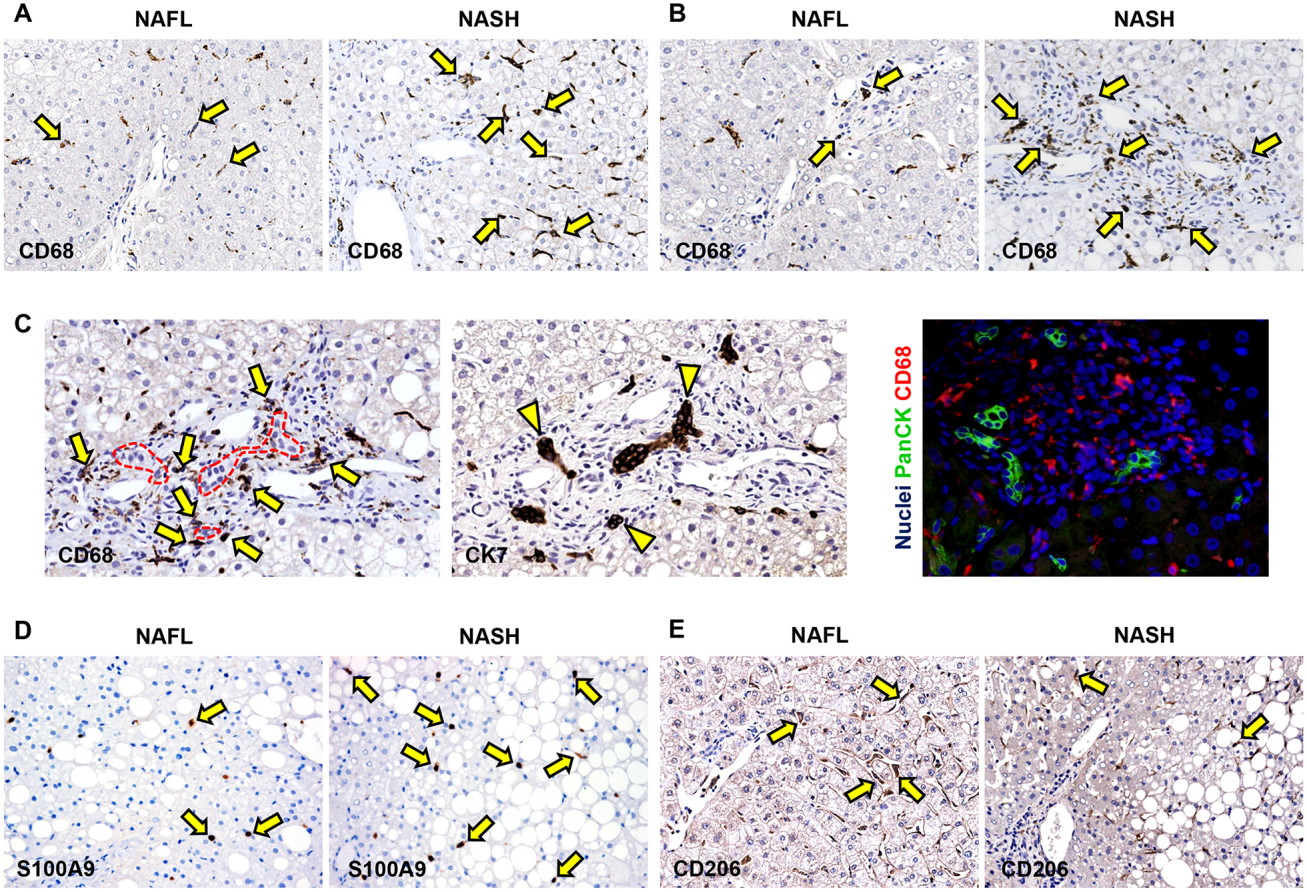
Macrophage subsets in pediatric NAFLD. A) Immunohistochemistry for CD68 in pediatric NAFLD biopsies. The number of CD68+ macrophages is increased (yellow arrows) in definite steatohepatitis (NASH) in comparison with not steatohepatitis (simple steatosis: NAFL). Original Magnification (OM) = 10x. B) Immunohistochemistry for CD68 in pediatric NAFLD biopsies. The number of portal CD68+ macrophages is increased (yellow arrows) in NASH in comparison with NAFL. OM = 10x. C) Immunohistochemistry for CD68 and Cytokeratin (CK) 7 in serial section shows that portal macrophages (arrows) are spatially associated with reactive ductules (dotted red line and arrowheads). These data were confirmed by immunofluorescence for Pan-CK and CD68. OM = 20x. D) Immunohistochemistry for S100A9 in pediatric NAFLD biopsies. The number of S100A9+ macrophages is increased (yellow arrows) in NASH in comparison with NAFL. Original Magnification (OM) = 10x. E) Immunohistochemistry for CD206 in pediatric NAFLD biopsies. The number of CD206+ macrophages is reduced (yellow arrows) in definite NASH in comparison with NAFL. Original Magnification (OM) = 10x.

When histological classification was taken in consideration (Fig 2A, Table 4), the number of total macrophages was higher in NASH (median: 28.80 [21.80, 36.50]) in comparison with NAFL biopsies (median: 17.30 [14.25, 23.60], p<0.02).

Then, portal and lobular CD68+ macrophages were separately counted; overall, the number of portal but not lobular macrophages was significantly increased in NAFLD in comparison with normal biopsies (Table 4). When histological classification was taken in consideration, biopsies with NASH showed an increased number of portal and lobular CD68+ macrophages in comparison with NAFL biopsies and normal samples (Fig 2A and 2B and Table 4). Moreover, biopsies with NAFL showed a higher number of portal but not lobular macrophages in comparison with normal specimens (Table 4). In NASH, CD68+ macrophages were found in close association with reactive ductules (Fig 2C).

Then, the number of macrophages expressing a marker of pro-inflammatory macrophages (S100A9) was evaluated (Fig 2D). Overall, in pNAFLD biopsies, the number of total S100A9+ macrophages was increased (median: 13.00 [7.25, 15.00]) in comparison with normal samples (median: 5.00 [4.00, 6.00], p<0.05, Table 4). When histological classification was taken in consideration (Fig 2D, Table 4), the number of total S100A9+ macrophages was higher in NASH (median: 15.00 [13.00, 15.00]) in comparison with NAFL biopsies (median: 7.00 [7.00, 10.50], p<0.02).

Then, portal and lobular S100A9+ macrophages were separately counted; overall, the number of portal and lobular macrophages was significantly increased in NAFLD in comparison with normal biopsies (Table 4). When histological classification was taken in consideration, biopsies with NASH showed an increased number of lobular S100A9+ macrophages in comparison with NAFL biopsies and normal samples (Fig 2D and Table 4) and a higher number of portal S100A9+ macrophages in comparison with normal samples. Moreover, biopsies with NAFL showed a higher number of S100A9+ portal and lobular macrophages in comparison with normal specimens (Table 4).

Finally, the number of macrophages expressing a marker of anti-inflammatory macrophages (CD206) was evaluated. Overall, in pNAFLD biopsies, the number of CD206+ macrophages was lower (median: 9.90 [7.67, 11.25]) in comparison with normal samples (median: 15.10 [12.50, 16.70], p<0.01).

When histological classification was taken in consideration (Fig 2E), the number of M2 macrophages was lower in NASH (median: 9.15 [7.32, 10.13]) in comparison with NAFL biopsies (median: 11.00 [9.91, 14.25], p<0.05).

Then, portal and lobular CD206+ macrophages were separately counted; overall, the number of lobular but not portal CD206+ macrophages was significantly reduced in NAFLD when compared with normal biopsies (Table 4). When histological classification was taken in consideration, biopsies with NASH showed a lower number of lobular CD206+ macrophages in comparison with NAFL biopsies and normal samples (**4**). Moreover, biopsies with NAFL showed a lower number of lobular but not portal CD206+ macrophages in comparison with normal specimens (Table 4).

The phenotypes of liver macrophages were correlated with the histo-pathological features of patients’ livers (Table 5). In particular, the number of CD68+, S100A9+, and CD206+ macrophages was correlated with NAS score (Table 5); moreover, the number of S100A9+ and CD206+ macrophages was correlated with steatosis and hepatocyte ballooning. When the number of portal macrophages was separately evaluated, it strongly correlated with the extension of portal fibrosis (r = 0.815, p<0.001).

**Table 5:** Correlations between total macrophage number and histo-pathological parameters in all pediatric NAFLD biopsies at baseline (N = 32).

	NAS	Steatosis	Ballooning	Lobular inflammation
**CD68+ Mϕ**	r = 0.59, **p = 0.044**	r = 0.51. p = 0.09	r = 0.46, p = 0.13	r = 0.48, p = 0.16
**S100A9+ Mϕ**	r = 0.79, **p< 0.001**	r = 0.71, **p = 0.002**	r = 0.64, **p = 0.002**	r = 0.31, p = 0.91
**CD206+ Mϕ**	r = - 0.89, **p = 0.003**	r = - 0,79, **p = 0.012**	r = - 0,81, **p = 0.030**	r = - 0.09, p = 0.58

Mϕ = macrophages; NAS = NAFLD Activity Score. *p values* < 0.05 are in **bold**.

### Effects of DHA treatment on HPC compartment and macrophage subsets

Twenty patients were treated with DHA for 18 months. Biopsies after the 18-month DHA treatment (T1) have been collected and compared with those at the baseline (T0). Variations in anthropometric and laboratory data were included in Table 2 and previous reports [14, 17]. As regard histo-pathological features, DHA treatment determined a significant reduction of liver steatosis, ballooning, and NAS (Table 2); furthermore, DR (median = 2.30 [2.30, 3.65]) was reduced at T1 in comparison with biopsies at the baseline (median = 5.00 [3.80, 6.15]; p<0.01). Modification of DR was strictly correlated with NAS score (r = 0.65; p<0.05) but not with other histo-pathological parameters (fibrosis, steatosis, ballooning, and lobular inflammation).

**Fig 3:**
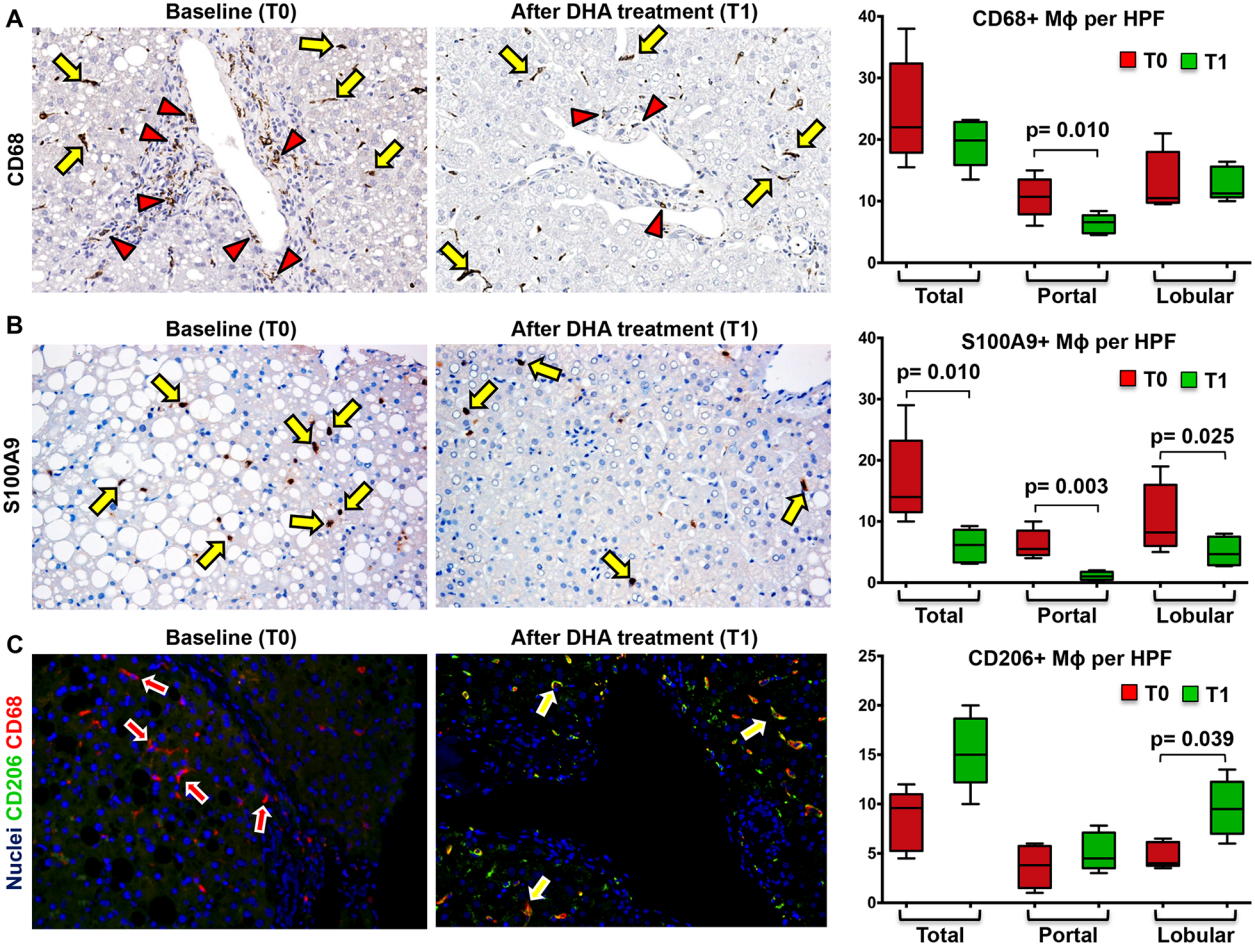
Docosahexaenoic acid (DHA) treatment modifies macrophage subsets in pediatric NAFLD. A) Immunohistochemistry for CD68 in pediatric NAFLD biopsies. The number of portal (red arrowheads) CD68+ macrophages (MΦs) is reduced after DHA treatment (T1) in comparison with baseline (T0) biopsies. No modifications in lobular CD68+ MΦ number (yellow arrows) are observed at T1. Original Magnification (OM) = 10x. B) Immunohistochemistry for S100A9 in pediatric NAFLD biopsies. The number of S100A9+ macrophages is reduced after DHA treatment (T1) in comparison with baseline (T0) biopsies. C) Double immunofluorescence for CD206 and CD68 in pediatric NAFLD biopsies. CD206+ macrophages are increased (yellow arrows) after DHA treatment (T1) in comparison with baseline (T0) biopsies. Original Magnification (OM) = 10x.

The number of total CD68+ macrophages (Fig 3A, Table 6) was not modified after DHA treatment (*Before Treatment*: Median = 22.00 [17.88, 32.35]; *After Treatment*: Median = 19.85 [15.88, 22.86]). Similarly, the number of lobular CD68+ macrophages was not modified after DHA treatment (*Before Treatment*: Median = 10.50 [9.75, 18.00]; *After Treatment*: Median = 11.25 [10.63, 15.60]); besides, the number of portal CD68+ macrophages was reduced after DHA treatment (*Before Treatment*: Median = 10.70 [7.87, 13.50]; *After Treatment*: Median = 6.60 [4.80, 7.70]; p< 0.02).

**Table 6:** Macrophage number and phenotype in liver biopsies obtained from pediatric patients affected by NAFLD at the baseline (T0) and at the end of DHA administration (T1).

	T0 (20)	T1 (20)	p
**CD68+ Mϕ**	22.00 [17.88, 32.35]	19.85 [15.88, 22.86]	0.22
**S100A9+ Mϕ**	14.00 [11.50, 23.20]	6.15 [3.30, 8.62]	**0.01**
**CD206+ Mϕ**	9.60 [5.25, 11.00]	15.00 [12.20, 18.66]	0.06
***Lo* CD68+ Mϕ**	10.50 [9.75, 18.00]	11.25 [10.63, 15.60]	0.84
***Lo* S100A9+ Mϕ**	8.20 [6.00, 16.00]	4.65 [2.85, 7.50]	**0.02**
***Lo* CD206+ Mϕ**	4.00 [3.75, 6.15]	9.50 [7.00, 12.25]	**0.04**
***Po* CD68+ Mϕ**	10.70 [7.87, 13.50]	6.60 [4.80, 7.70]	**0.01**
***Po*S100A9+ Mϕ**	5.50 [4.50, 8.50]	1.00 [0.45, 1.75]	**0.00**
***Po* CD206+ Mϕ**	3.80 [1.50, 5.75]	4.50 [3.50, 7.11]	0.31

Data are indicated as Median [25^th^ percentile, 75^th^ percentile]. Mϕ = macrophages; lo = lobular; po = portal. T0 = baseline; T1 = at the end of treatment. *p values* < 0.05 are in **bold**.

The number of total S100A9+ macrophages (Fig 3B, Table 6) was reduced after DHA treatment (*Before Treatment*: Median = 14.00 [11.50, 23.20]; *After Treatment*: Median = 6.15 [3.30, 8.62]; p = 0.010). Moreover, the number of lobular S100A9+ macrophages was reduced after DHA treatment (*Before Treatment*: Median = 8.20 [6.00, 16.00]; *After Treatment*: Median = 4.65 [2.85, 7.50], p = 0.024); similarly, the number of portal S100A9+ macrophages was reduced after DHA treatment (*Before Treatment*: Median = 5.50 [4.50, 8.50]; *After Treatment*: Median = 1.00 [0.45, 1.75]; p = 0.0038).

**Fig 4:**
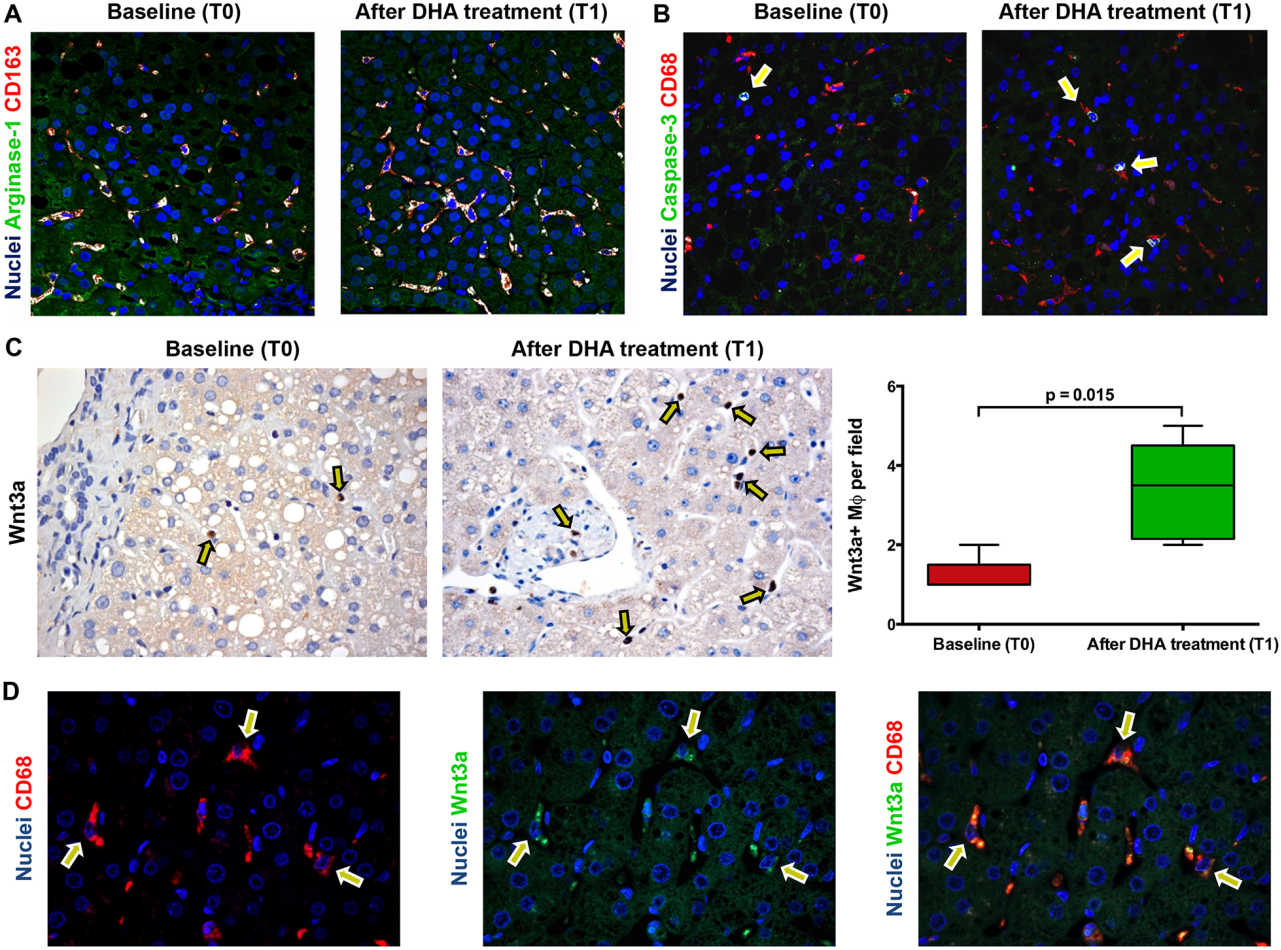
Phenotype, apoptosis and wnt3a expression in macrophages are modified by docosahexaenoic acid (DHA) treatment in pediatric NAFLD. A) Immunofluorescence for Arginase-1 and CD163 confirms the increase of macrophages (MΦs) with an anti-inflammatory phenotype after DHA treatment (T1) in comparison with baseline biopsies (T0). Original Magnification (OM) = 20x. B) Immunofluorescence for Caspase-3 and CD68 shows the increased macrophage apoptosis at T1. OM = 20x. C) Immunohistochemistry for Wnt3a in pediatric NAFLD biopsies. The number of macrophages expressing Wnt3a is increased after DHA treatment (arrows) in comparison with baseline biopsies. OM = 10x. D) Immunofluorescence for Wnt3a and CD68 in biopsies after DHA confirms the Wnt3a expression in MΦs (arrows). OM = 10x.

On the other hand, the number of lobular (but not total or portal) CD206+ macrophages (Fig 3C, Table 6) was increased after DHA treatment (*Before Treatment*: Median = 4.00 [3.75, 6.15]; *After Treatment*: median = 9.50 [7.00, 12.25]; p<0.05; Fig 3A and 3B). The increase of lobular anti-inflammatory macrophages spectrum was further confirmed counting the number of double positive CD163 and Arginase-1 macrophages (*Before Treatment*: median = 8.00 [5.17, 10.50]; *After Treatment*: median = 13.00 [9.66, 13.33]; p<0.05) (Fig 4A). Accordingly, the number of lobular CD206+ macrophages was correlated with the number of CD163+/Arginase1+ macrophages (r = 0.720; p = 0.023).

DHA treatment caused the increased number of apoptotic macrophages (*Before Treatment*: median = 9.67 [7.17, 10.33]; *After Treatment*: median = 11.67 [10, 13.33]; p< 0.05; Fig 4B). The number of apoptotic macrophages is correlated with the number of CD206+ (r = 0.650; p< 0.05) and CD163+/Arginase1+ macrophages (r = 0.729; p = 0.021, Table 6).

As regard histo-morphological parameters, the number of S100A9+ macrophages was directly correlated with NAS and DR extension and the number of CD206+ macrophages was inversely correlated with NAS, hepatocyte steatosis and lobular inflammation (Table 7). Finally, as regard clinical parameters, S100A9+ macrophages and DR extensions are correlated with serum levels of pro-inflammatory cytokines such as TNFα, IL-6, and IL-1B (Table 8).

**Table 7:** Correlations between modifications of macrophage phenotype, histo-pathological features, ductular reaction and serum inflammatory cytokine levels after DHA treatment.

	NAS	Steatosis	Ballooning	LI	Wnt3a+ Mϕ	Apoptotic Mϕ
**CD68+ Mϕ**	r = 0.11, p = 0.74	r = 0.18, p = 0.60	r = 0.12, p = 0.71	r = 0.20, p = 0.57	r = - 0.12, p = 0.73	r = - 0.03, p = 0.94
**S100A9+ Mϕ**	r = 0.71, **p = 0.03**	r = 0.61, p = 0.06	r = 0.10, p = 0.77	r = 0.43, p = 0.22	r = - 0.73, **p = 0.02**	r = - 0.44, p = 0.20
**CD206+ Mϕ**	r = - 0.90, **p< 0.01**	r = - 0,73, **p = 0.02**	r = - 0,18, p = 0.62	r = - 0.68, **p = 0.03**	r = 0.88, **p< 0.01**	r = 0.65, **p = 0.04**

Mϕ = macrophages; NAS = NAFLD Activity Score; LI = lobular inflammation; *p values* < 0.05 are in **bold**.

**Table 8:** Correlations between modifications of macrophage phenotype, histo-pathological features, ductular reaction and serum inflammatory cytokine levels after DHA treatment.

	NAS	WNT3a	S100A9	TNFα	IL-6	IL-1B
**DR**	r = 0.65, **p = 0.04**	r = - 0.68, **p = 0.04**	r = 0.71, **p = 0.03**	r = 0.65, **p = 0.04**	r = 0.83, **p = 0.005**	r = 0.782, **p = 0.01**
**S100A9+ Mϕ**	r = 0.71, **p = 0.03**	r = - 0.73, **p = 0.02**	r = 1.00, p = .	r = 0.83, **p< 0.01**	r = 0.74, **p = 0.02**	r = 0.81, **p< 0.01**

Mϕ = macrophages; NAS = NAFLD Activity Score; DR = Ductular Reaction; TNF = Tumor Necrosis Factor; IL = interleukin. *p values* < 0.05 are in **bold**.

### DHA treatment induced Wnt pathway activation in pNAFLD

Before DHA treatment (Fig 4), the expression of Wnt3a by macrophages in pNAFLD was low with rare positive macrophages within liver parenchyma (Median = 1.00 [1.00, 1.50]). After DHA treatment, the number of Wnt3a+ macrophages was significantly increased (Median = 3.50 [2.15, 4.50]) in comparison with biopsies before the treatment (p<0.02).

Interestingly, the increasing of Wnt3a+ macrophages after DHA treatment was directly correlated with the number of CD206+ macrophages (r = 0.88, p<0.01, Table 7) and inversely correlated with the number of S100A9+ macrophages (r = -0.73, p<0.05, Table 7), NAS score (r = -0.778, p<0.01), steatosis (r = -0.691, p<0.05), DR extension (r = -0.68, p<0.05), and serum levels of pro-inflammatory cytokines (TNF-alpha: r = 0.641, p<0.05; IL-1B: r = 0.735, p<0.02).

**Fig 5:**
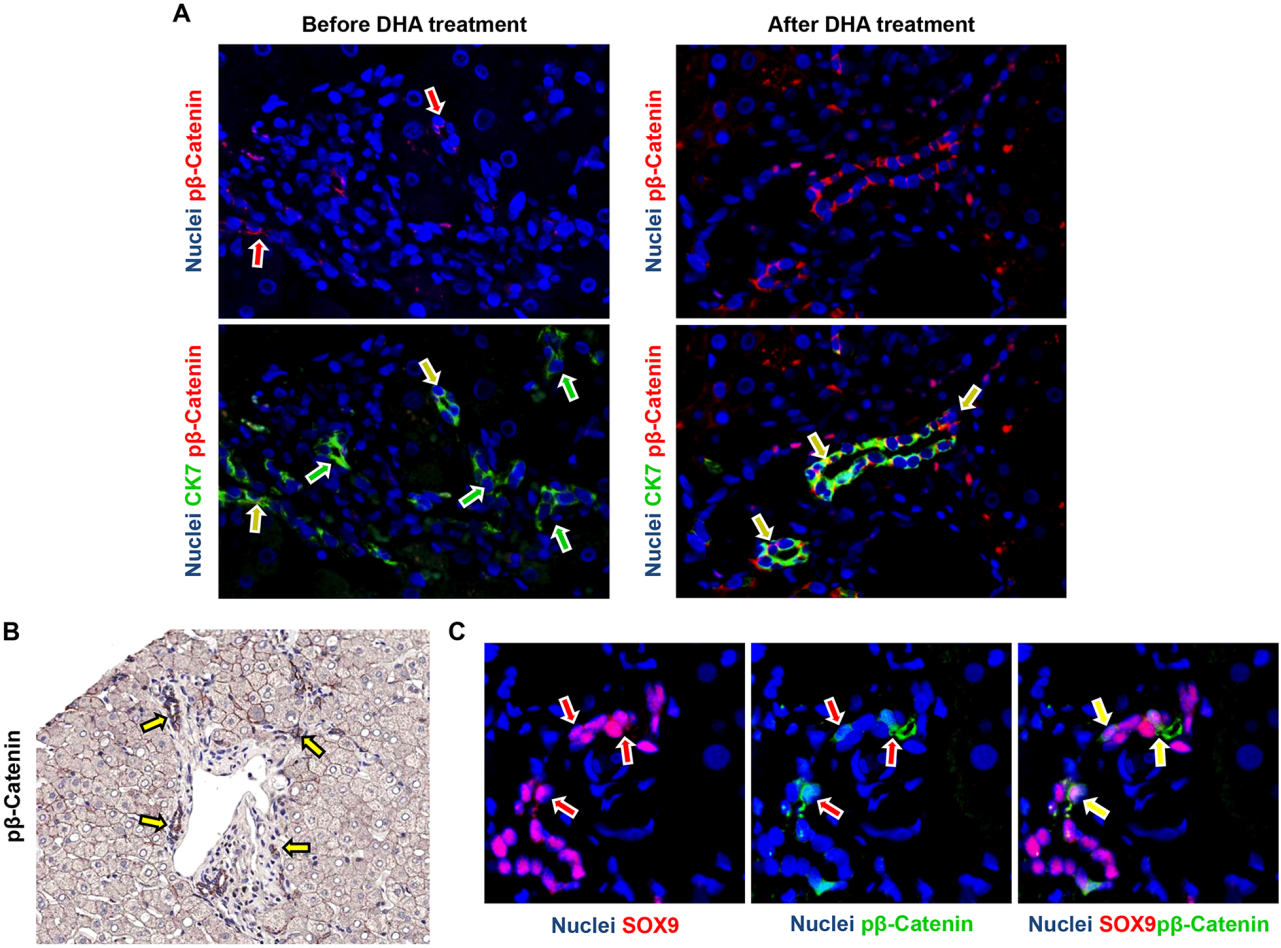
Modification of phosphorylated (p) β-catenin expression in ductular reaction and associated hepatic progenitor cells (HPCs) in pediatric NAFLD after docosahexaenoic acid (DHA) treatment. A) Immunofluorescence for phosphorylated (p) β-catenin and Cytokeratin(CK)7 in pediatric NAFLD biopsies. After DHA treatment, the number of pβ-catenin positive cells within reactive ductules is increased (yellow arrows); green arrows indicate CK7+ ductular cells not expressing pβ-catenin. Original Magnification (OM) = 20x. B) Immunohistochemistry for pβ-catenin in pediatric NAFLD biopsies after DHA treatment confirms the expression of pβ-catenin by ductular reaction (arrows). OM = 10x. C) Immunofluorescence for phosphorylated (p) β-catenin and SOX9 in pediatric NAFLD biopsies. pβ-Catenin+ cells within reactive ductules were positive for the progenitor cell marker SOX9 (arrows). OM = 40x.

In pNAFLD biopsies at the baseline, the percentage of pβ-Catenin+ HPCs (Fig 5) was higher in NASH (Median = 11.90 [6.42, 13.65]) in comparison with NAFL biopsies (Median = 5.20 [1.50, 10.85]); moreover, when biopsies were sub-divided according to the presence of EpCAM+ hepatocytes, biopsies with EpCAM+ hepatocytes contained more pβ-Catenin+ HPCs (Median = 12.30 [12.00, 14.78]) in comparison with those without (Median = 6.20 [3.25, 11.53]). After DHA treatment, the percentage of pβ-Catenin+ HPCs was significantly increased (Median = 50 [20, 55]) in comparison with biopsies before the treatment (p<0.01). Given the differences in term of DR extension among examined biopsies, pβ-Catenin positivity was further calculated as the ratio between the extension of positive DR and the total DR extension. Accordingly, DHA treatment determined a marked increase of pβ-Catenin+ cells within DR (Median = 2.70 [1.04, 3.93]) in comparison with biopsies at the baseline (Median = 0.78 [0.28, 1.27]; p<0.02). pβ-Catenin+ cells within reactive ductules were positive for the progenitor cell marker SOX9 (Fig 5C). The increasing of pβ-Catenin+ HPCs was directly correlated with the number of Wnt3a+ macrophages (r = 0.869, p<0.001) and the presence of EpCAM+ hepatocytes (r = 0.620, p<0.05) and inversely correlated with NAS score (r = -0.669, p<0.05) and DR extension (r = -0.637, p<0.05).

## Discussion

The main findings of the present study indicate that in pNAFLD: i) the progression towards NASH is characterized by the modification of the resident macrophage pool; ii) in pNAFLD, the macrophage activation is correlated with NAS, DR, and degree of portal fibrosis; iii) the administration of DHA in pediatric patients is able to modulate macrophage activation by reducing total liver macrophages and by modifying the number of macrophages with a pro-inflammatory and anti-inflammatory phenotypes; iv) the modulation of macrophage activation by DHA treatment is correlated with the reduction of serum level of inflammatory cytokines, with increased macrophage apoptosis, and with the up-regulation of macrophage Wnt3a expression; and v) the Wnt3a expression by macrophages is correlated with β-catenin phosphorylation in ductular reaction.

In adult NAFLD, as a consequence of hepatocyte death, liver macrophages can accumulate large amounts of lipids, transform into foam cells and drive progression towards steatohepatitis [4]. Our results indicated that pNAFLD biopsies were characterized by the modification of macrophage pool. In accordance with recent guidelines on macrophage nomenclature, we used different markers (CD206, S100A9, Arginase-1, CD163) to define activation states on the anti-inflammatory or pro-inflammatory spectrum range [5, 34]. In our setting, simple steatosis (NAFL) is characterized by an initial increase of portal (but not lobular or total) macrophage number and a modification of lobular macrophage phenotype (pro-inflammatory rather than anti-inflammatory); then, the progression to NASH is characterized by a further increase of overall macrophage population and the predominance of macrophages with a pro-inflammatory rather than an anti-inflammatory phenotype.

The release of macrophage-derived mediators could contribute to inflammation and fibrogenesis [4, 35]; in adult NAFLD, portal macrophage infiltration is strongly correlated with HPC activation and portal fibrosis [36]. In pNAFLD, HPC activation takes place as a consequence of hepatocyte cell cycle arrest and apoptosis [11, 37]. Proliferating HPCs are able to determine the local activation of fibrogenic cells, thus inducing collagen-I deposition and establishing a pro-fibrogenic loop [38, 39]. In parallel, our data showed that, in pNAFLD, progressive portal macrophage accumulation is correlated with portal fibrosis; portal macrophages were spatially close to DR, which was, in turn, correlated with portal fibrosis. These observations seem to suggest the cross-talk between macrophages and HPCs as a main driver of portal fibrosis in pNAFLD.

To test the hypothesis that macrophage activation is associated with the progression towards NASH and HPC activation, liver biopsies from patients treated with DHA for 18-months were examined. In this clinical setting, the treatment with algae DHA improved liver histo-pathology (steatosis, NAS) and was able to reduce the serum ALT levels and triglycerides [14, 15]. Short-term DHA treatment seems to be not sufficient to achieve clinical amelioration, thus suggesting that not less than 12 months of therapy are needed to obtain both the anti-steatotic and anti-infllammatory effects [40]. In an experimental model, dietary DHA suppressed hepatic markers of oxidative stress, inflammation and fibrosis [41]. DHA modulates G protein-coupled receptor 120 (GPR120), acting as a negative feedback signal on NF-κB phosphorylation induced by Toll Like Receptors and TNF-α cascade on macrophages [16, 42]. GPR120 could further decrease pro-inflammatory and increase anti-inflammatory gene expression in macrophages [4].

Notably, after DHA treatment, the number of CD68+ macrophages was unchanged; however, we observed a reduction of pro-inflammatory (S100A+) macrophages and an increase of lobular macrophages with an anti-inflammatory phenotype (CD206+, CD163+, Arginase-1+); these modifications correlated with the improvement of histo-pathological parameters such as NAS, hepatocyte staetosis and lobular inflammation. Furthermore, DHA determined the increasing of apoptotic macrophages; interestingly, the number of apoptotic macrophages directly correlated with the number of CD206+ macrophages, thus suggesting that the apoptosis of pro-inflammatory macrophage could have a role in the determination of macrophage activation state induced by DHA treatment.

As regards clinical parameters, the modification of macrophage activation state induced by DHA supplementation correlated with the reduction of serum levels of pro-inflammatory cytokines. Given the emerging role of the liver as a source of inflammatory mediators in the course of metabolic syndrome and in the progression of atherosclerosis [43, 44], this association needs further attention and should be evaluated as a clinical outcome in larger studies.

Previous experimental studies in rodents clearly indicated that the efficient phagocytosis of hepatocyte debris determines the induction of Wnt3a expression in macrophages and the up-regulation of Wnt signaling in nearby HPCs, promoting their specification to hepatocytes [8]. In the present manuscript, Wnt3a-positive macrophages significantly increased after DHA treatment in correlation with phenotype changes; the presence of Wnt3a-positive macrophages was correlated with β-catenin phosphorylation in SOX9+ HPCs within DR; moreover, pβ-catenin expression in HPC correlated with the presence of scattered EpCAM+ hepatocytes. In human pathologies, EpCAM+ hepatocytes have been shown to represent the progeny of stem/progenitor cells within bile ductules [29, 30] participating, in part, in the repopulation of liver parenchyma by the bud sequence [29, 45].

Although the present study is not mechanistic, our results are in keeping with previous experimental evidence on the relationship between macrophage activation and progenitor cell response [8] and suggest the possibility that this cross-talk can have a role in the course of pNAFLD.

In conclusion, macrophage infiltration and activation seem to have a key role in the progression toward NASH in pNAFLD; the induction of the pro-inflammatory macrophage activation could trigger HPC proliferation and the activation of pro-fibrogenetic loop; the administration of dietary DHA influences macrophage activation and could have a role in the modulation of HPC response by Wnt3a production.
